# Supplementary material for: Analysis of PPARγ Signaling Activity in Psoriasis
Source: Int J Mol Sci. 2021 Aug 10;22(16):8603. doi: 10.3390/ijms22168603 (PMC8395241; doi:10.3390/ijms22168603)
Supplement: Supplementary file 1 [file ijms-22-08603-s001.zip › Supplemental materials_Analysis of PPARg signaling activity in psoriasis/Pathway models/Models images and html files/Differentiation of psoriatic T cells/11745.html]

TYK2


# Protein TYK2

|  |  |
| --- | --- |
| URN | urn:agi-llid:7297 |
| Total Entities | 0 |
| Connectivity | 4875 |
| Name | TYK2 |
| Description | tyrosine kinase 2 |
| Notes | This gene encodes a member of the tyrosine kinase and, more specifically, the Janus kinases (JAKs) protein families. This protein associates with the cytoplasmic domain of type I and type II cytokine receptors and promulgate cytokine signals by phosphorylating receptor subunits. It is also component of both the type I and type III interferon signaling pathways. As such, it may play a role in anti-viral immunity. A mutation in this gene has been associated with hyperimmunoglobulin E syndrome (HIES) - a primary immunodeficiency characterized by elevated serum immunoglobulin E. [provided by RefSeq, Jul 2008] |
| Primary Cell Localization | Cytoplasm |
| Class | Protein kinase |
| ObjectType | Protein |

---

|  |  |
| --- | --- |
| Pathway | Thrombopoietin Receptors Signaling in Platelet Maturation |
|  | CD8+ T-Cell Activation |
|  | Th1-Cell Differentiation |
|  | AHR Signaling in Th17 Cells Function |
|  | Th17-Cell Differentiation |
|  | Plasmin Effects in Inflammation |
|  | AHR in Intestinal Cell Antimicrobial Barrier Maintenance |
|  | Mast-Cell Activation without Degranulation |
|  | anti-Inflammatory Function of Macrophage M2 Lineage |
|  | AXL Receptor Inhibits Macrophages and Dendritic Cells Function |
|  | Positive Acute Phase Proteins Synthesis |
|  | Vascular Endothelial Cell Activation by Cytokines |
|  | Vascular Endothelial Cell Activation by Blood Coagulation Factors |
|  | Vascular Endothelial Cell Activation by Growth Factors |
|  | IL6 Expression Targets -> Nociception |
|  | Th1-Cells Activation and Proliferation in Atherosclerosis |
|  | Lymphocyte Mediated Myocardial Injury in Myocarditis |
|  | SOCS in Negative Feedback Regulation in Myocarditis |
|  | Atopic Dermatitis |
|  | T-Cells Differentiation Block in Psoriasis |
|  | IL22 Induces Keratinocyte Proliferation in Psoriasis |
|  | Dendritic Cell Activation in Systemic Lupus Erythematosis |
|  | Th17-Cell Function in Systemic Lupus Erythematosus |
|  | Th1-Cell Function in Systemic Lupus Erythematosus |
|  | B-Cell Activation in Systemic Lupus Erythematosus |
|  | Vitiligo |
|  | T-Cell Cytotoxic Response against Melanocytes in Vitiligo |
|  | Humoral Immunity in Vitiligo |
|  | Toll-like Receptors in beta-Cell |
|  | Adipokines and Cytokines in Insulin Resistance in Skeletal Muscles |
|  | CD4+T-Cell Response in Celiac Disease |
|  | Th1-Cell Activation in Crohn's Disease |
|  | Th17-Cell Activation in Crohn's Disease |
|  | Genes with Susceptibility for Crohn's Disease and Ulcerative Colitis |
|  | Leukocyte Migration toward the Endothelial Cell in Ulcerative Colitis |
|  | IL6/IGF1/VEGFA Signaling in Multiple Myeloma |
|  | Multiple Myeloma |
|  | Eosinophil Survival in Asthma |
|  | Il17 Signaling Related Neutrophilia in Asthma |
|  | Periostin (POSTN) Production by Airway Epithelium in Asthma |
|  | Airway Smooth Muscle Cell High Rate Proliferation |
|  | Goblet-Cell Related Mucus Secretion in Asthma |
|  | Club-Cell Role in Asthma |
|  | Th17-Cell Differentiation in Asthma |
|  | Mucin Production in Goblet Airway Epithelial Cells |
|  | Dendritic Cell Dysfunction in Psoriatic Arthritis |
|  | Th17-Cell and Th1 Immune Responsein Psoriatic Arthritis |
|  | Keratinocyte Activation in Psoriatic Arthritis |
|  | B-Cells Function in Systemic Scleroderma |
|  | Th17-Cell Numbers Reduction in HIV |
|  | Diabetes Induced Periodontitis |
|  | Genes with Mutations Associated with Psoriatic Arthritis |
|  | Glioma Invasion Signaling |
|  | IL6/IL12 Signaling Activates Immune System in Multiple Sclerosis |
|  | Proteins Involved in Endometriosis |
|  | IL6/JAK/STAT3 Signaling in Cholesteatoma |
|  | Cholesteatoma |
|  | Neurotrophic Factor Deprivation in Retinal Ganglion Cell Death |
|  | Cytokines Trigger Otitis Media |
|  | Mucin Hyperproduction in Goblet and Mucous Cells |
|  | CLCF1 Expression Targets |
|  | CTF1 Expression Targets |
|  | CSF3 Expression Targets |
|  | LIF Expression Targets |
|  | CNTF Expression Targets |
|  | F2 -> STAT1/NF-kB Expression Targets |
|  | PLG -> STAT1/NF-kB Expression Targets |
|  | GH2/GHR Expression Target |
|  | GH1/GHR -> STAT Expression Targets |
|  | CSH1/GHR Expression Targets |
|  | Receptors and Adaptor Proteins Activated in Cancer |
|  | STAT3 and NFkB Activate Inflammation-Induced Tumorigenesis |
|  | IL23A and IL17A Provoke Cancer-Associated Inflammation |
|  | STAT3 Inhibits Maturation of Dendritic Cells and Facilitates Cancer Progression |
|  | STAT3 Facilitates the Function of Treg Cells and Cancer Progression |
|  | IL12 Antitumor Effect is Blocked in Cancer |
|  | IL13/STAT6/ZEB1 Signalling Promotes Cancer Metastatic Progression |
|  | N2 Neutrophils in Tumor-Promoting Inflammation and Tumor Progression |
|  | IDO1 in Cancer Immune Escape |
|  | IFNLR1-> STAT/NFKB Signaling |
|  | IFNW1/IFNR Expression Target |
|  | IFNA1/IFNR Expression Targets |
|  | IFNB1/IFNR Expression Targets |
|  | IL22R -> STAT3 Signaling |
|  | IL10R -> STAT Signaling |
|  | IL13R -> STAT Signaling |
|  | IL6R -> STAT Signaling |
|  | IL12R -> STAT Signaling |
|  | IL12R -> NF-kB/NFATC Signaling |
|  | IL13 Expression Targets |
|  | IL12B Expression Targets |
|  | IL6 Expression Targets |
|  | IL10 Expression Targets |
|  | IL22 Expression Targets |
|  | UrokinaseR -> ELK/SRF Signaling |
|  | UrokinaseR -> STAT Signaling |
|  | OSM/IL6ST/LIFR Expression Targets |
|  | CSF2 -> STAT Signaling |
|  | PRL/GHR -> STAT Expression Targets |
|  | CSF1 -> STAT Expression Targets |
|  | PAF Expression Targets |
|  | Thrombopoietin -> STAT Expression Targets |
|  | PLAU -> STAT1 Expression Targets |
|  | PLAU -> ELK-SRF/AP-1 Expression Targets |
|  | VEGFA -> STAT Expression Targets |
|  | VEGFR -> STAT Signaling |
|  | PPAR Psoriasis |
|  | Model of PPARG signaling in psoriasis |
|  | New Pathway (3) |
|  | dsRNA induces innate immunity and INFs in alveolar cells |
|  | IFN signaling in alveolar cells |
|  | Dendritic Cell Activation in COVID19 |
|  | 1\_Differentiation of psoriatic T cells |
|  | 2\_Interleukin-17 and interleukin-22 signaling in psoriasis |
|  | 2\_The role of endothelial cells in pyelonephritis |
|  | 3\_Role of neurotrophic factor deprivation in retinal ganglion cell death |
|  | 3\_2\_Regulation of airway surface liquid (ASL) thickness |
|  | 3\_1\_Mucus accumulation in asthma |
|  | 2\_2\_Eosinophilia and neutrophilia in asthma: eosinophil apoptosis |
|  | 4\_Mucin hyperproduction in goblet and mucous cells in COPD |
|  | 1\_Defects in response to pathogens in the gut promotes inflammation in Crohn's disease |
|  | 3\_Leukocyte migration toward the endothelial cells in the intestine microvasculature |
|  | 4\_1\_Polymorphisms associated with inflammatory bowel diseases |
|  | 1\_Antigen-presenting cells promote islet dysfunction and immune system activation in T1D |
|  | 2\_Defective tolerance of autoreactive T-cell in T1D |
|  | Differentiation of psoriatic T cells |
|  | Interleukin-17 and interleukin-22 signaling in psoriasis |

---

|  |  |
| --- | --- |
| Group | Genes Associated with Systemic Lupus Erythematosus |
|  | Genes with Mutations Associated with Inflammatory Bowel Diseases |
|  | Genes with Mutations Associated with Psoriatic Arthritis |
|  | Genes with Mutations Associated with Endometriosis |

---

|  |  |
| --- | --- |
| Source | curated |
|  | GO component |

---

|  |  |
| --- | --- |
| MedScan ID | 7297 |
|  | 650588 |
|  | 298706 |

---

|  |  |
| --- | --- |
| LocusLink ID | 7297 |
|  | 54721 |
|  | 100361294 |
|  | 298706 |
|  | 512484 |

---

|  |  |
| --- | --- |
| Alias | Non-receptor tyrosine-protein kinase TYK2 |
|  | protein-tyrosine kinase tyk2 |
|  | JTK1 |
|  | JTK I |
|  | TYK2 kinase |
|  | tyrosine kinase II |
|  | LOC115716 |
|  | protein kinase TYK2 |
|  | LOC100361294 |
|  | tyrosine kinase 2 |
|  | TYK2\_HUMAN |
|  | TYK2 |
|  | IMD35 |
|  | tyrosine kinase TYK2 |
|  | protein-tyrosine kinase tyk2 |

---

|  |  |
| --- | --- |
| EC Number | 2.7.10.2 |
|  | 2.7.10.1 |
|  | 2.7.1.112 |

---

|  |  |
| --- | --- |
| GO ID | 0005524 |
|  | 0005131 |
|  | 0004715 |
|  | 0004713 |
|  | 0031702 |
|  | 0019221 |
|  | 0035722 |
|  | 0038155 |
|  | 0070106 |
|  | 0035556 |
|  | 0018108 |
|  | 0006468 |
|  | 0060337 |
|  | 0005737 |
|  | 0005856 |
|  | 0005829 |
|  | 0070062 |
|  | 0016020 |
|  | 0005634 |
|  | 0016301 |
|  | 0000166 |
|  | 0004672 |
|  | 0016740 |
|  | 0016310 |
|  | 0045087 |
|  | 0051427 |
|  | 0031234 |
|  | 0016477 |
|  | 0016772 |
|  | 0038083 |
|  | 0007243 |
|  | 0007169 |
|  | 0005102 |
|  | 0030154 |
|  | 0060338 |
|  | 0042127 |
|  | 0004718 |
|  | 0005515 |
|  | 0007242 |
|  | 0004674 |

---

|  |  |
| --- | --- |
| KEGG ID | hsa:7297 |
|  | mmu:54721 |
|  | rno:100361294 |

---

|  |  |
| --- | --- |
| MedLine Reference | 10409622 |
|  | 10502458 |
|  | 10531356 |
|  | 10542297 |
|  | 10673353 |
|  | 10899310 |
|  | 10933219 |
|  | 10995743 |
|  | 11399767 |
|  | 11430697 |
|  | 12477932 |
|  | 12554654 |
|  | 12719789 |
|  | 12960323 |
|  | 1386289 |
|  | 14500680 |
|  | 15489334 |
|  | 15883164 |
|  | 15944400 |
|  | 16239216 |
|  | 16551269 |
|  | 17011030 |
|  | 17384181 |
|  | 17548050 |
|  | 2156206 |
|  | 2216457 |
|  | 2247464 |
|  | 7526154 |
|  | 7579387 |
|  | 7589562 |
|  | 7629131 |
|  | 8125298 |
|  | 8432525 |
|  | 8550573 |
|  | 8702790 |
|  | 8780698 |
|  | 9108397 |
|  | 9196040 |
|  | 9305869 |
|  | 9342324 |
|  | 9373149 |
|  | 9374471 |
|  | 9388212 |
|  | 9519782 |
|  | 9633884 |
|  | 9733772 |
|  | 9822675 |
|  | 10349636 |
|  | 11042159 |
|  | 11070173 |
|  | 11070174 |
|  | 11076861 |
|  | 11217851 |
|  | 11313413 |
|  | 11313414 |
|  | 11437454 |
|  | 11877284 |
|  | 12391177 |
|  | 12466851 |
|  | 12517976 |
|  | 12594853 |
|  | 12679810 |
|  | 14500783 |
|  | 14617019 |
|  | 14662340 |
|  | 14768057 |
|  | 15226272 |
|  | 15254900 |
|  | 15488705 |
|  | 15491990 |
|  | 15578097 |
|  | 15782199 |
|  | 16141072 |
|  | 16141073 |
|  | 16148148 |
|  | 16601124 |
|  | 16751369 |
|  | 16982690 |
|  | 17048270 |
|  | 17078929 |
|  | 17372006 |
|  | 17395783 |

---

|  |  |
| --- | --- |
| Organism | Homo sapiens {Organism urn:agi-taxid:9606} |
|  | Mus musculus {Organism urn:agi-taxid:10090} |
|  | Rattus norvegicus {Organism urn:agi-taxid:10116} |
|  | Mus musculus |
|  | Homo sapiens |
|  | Rattus norvegicus |
|  | Bos taurus |

---

|  |  |
| --- | --- |
| Mouse chromosome position | 9 |
|  | 9 A3 |

---

|  |  |
| --- | --- |
| OMIM ID | 176941 |
|  | 611521 |

---

|  |  |
| --- | --- |
| Rat chromosome position | 8q13 |
|  | 8 |

---

|  |  |
| --- | --- |
| Hugo ID | 12440 |
|  | HGNC:12440 |

---

|  |  |
| --- | --- |
| Human chromosome position | 19p13.2 |

---

|  |  |
| --- | --- |
| Swiss-Prot Accession | A0A024R7E4 |
|  | P29597 |
|  | P29597.3 |
|  | E9QJS1 |
|  | Q9R117 |
|  | Q9R117.3 |
|  | D3ZD03 |
|  | Q6QB10 |
|  | Q96CH0 |
|  | O88431 |
|  | Q3TXE3 |
|  | Q52KQ2 |
|  | Q8VE41 |
|  | Q9R117.2 |
|  | Q53HA9 |
|  | Q2M4G5 |
|  | Q3TDN4 |
|  | Q3TIS9 |
|  | Q3U447 |
|  | TYK2\_HUMAN |
|  | INGR1\_HUMAN |

---

|  |  |
| --- | --- |
| PIR ID | S12127 |

---

|  |  |
| --- | --- |
| GenBank ID | NC\_000019 |
|  | XR\_002958353 |
|  | XR\_001753750 |
|  | XR\_001753751 |
|  | XR\_001753752 |
|  | XM\_011528245 |
|  | XP\_011526547 |
|  | XM\_011528246 |
|  | XP\_011526548 |
|  | XM\_011528247 |
|  | XP\_011526549 |
|  | XM\_011528251 |
|  | XP\_011526553 |
|  | XM\_011528249 |
|  | XP\_011526551 |
|  | NM\_003331 |
|  | NP\_003322 |
|  | NG\_007872 |
|  | AC011557 |
|  | AY549314 |
|  | AAS37680 |
|  | CH471106 |
|  | EAW84099 |
|  | EAW84100 |
|  | KT584692 |
|  | AK222672 |
|  | BAD96392 |
|  | AK303627 |
|  | BAG64635 |
|  | AK308360 |
|  | AK308379 |
|  | BC014243 |
|  | AAH14243 |
|  | CR995139 |
|  | DA864169 |
|  | X54637 |
|  | CAA38449 |
|  | P29597 |
|  | NC\_000075 |
|  | NM\_018793 |
|  | NP\_061263 |
|  | NM\_001205312 |
|  | NP\_001192241 |
|  | XR\_001778942 |
|  | XM\_006510492 |
|  | XP\_006510555 |
|  | XM\_011242579 |
|  | XP\_011240881 |
|  | XM\_006510493 |
|  | XP\_006510556 |
|  | XM\_006510494 |
|  | XP\_006510557 |
|  | AC163637 |
|  | AF052607 |
|  | AAC34580 |
|  | AY902350 |
|  | AAY21060 |
|  | CH466522 |
|  | EDL25159 |
|  | EDL25160 |
|  | AF173032 |
|  | AAD49423 |
|  | AK134154 |
|  | AK154438 |
|  | BAE32587 |
|  | AK155923 |
|  | AK159303 |
|  | BAE34973 |
|  | AK165483 |
|  | AK167726 |
|  | BAE39767 |
|  | AK170107 |
|  | BAE41567 |
|  | AK170940 |
|  | AK171609 |
|  | AK195177 |
|  | AK195939 |
|  | AK199641 |
|  | AK199646 |
|  | AK206451 |
|  | AK206664 |
|  | AK217061 |
|  | BC019789 |
|  | AAH19789 |
|  | BC053021 |
|  | BC094240 |
|  | AAH94240 |
|  | Q9R117 |
|  | NC\_005107 |
|  | XR\_593758 |
|  | XR\_593759 |
|  | XM\_006242586 |
|  | XP\_006242648 |
|  | XM\_006242585 |
|  | XP\_006242647 |
|  | NM\_001257347 |
|  | NP\_001244276 |
|  | XR\_001839127 |
|  | AC\_000076 |
|  | AABR07069438 |
|  | AAHX01053581 |
|  | XP\_005260106 |
|  | ABM83618 |
|  | XM\_005260049 |
|  | XR\_356112 |
|  | XR\_356114 |
|  | XR\_356113 |
|  | AAHY01076444 |
|  | XM\_006242588 |
|  | XM\_006242587 |
|  | XM\_006510495 |
|  | AABR06053665 |
|  | AC\_000151 |
|  | AC\_000031 |
|  | NC\_018930 |
|  | ABBA01029934 |
|  | ABBA01029932 |
|  | ABBA01029933 |
|  | XP\_011526554 |
|  | XP\_006510558 |
|  | XP\_011526550 |
|  | AMYH02035492 |
|  | XP\_006242649 |
|  | XP\_011526552 |
|  | AMYH02035491 |
|  | ABM86881 |
|  | XM\_011528248 |
|  | DQ892692 |
|  | XM\_011528252 |
|  | XM\_011528250 |
|  | DQ895955 |
|  | XP\_006242650 |
|  | NT\_011295 |
|  | NW\_001838483 |
|  | NW\_927195 |
|  | AC\_000062 |
|  | Q53HA9 |
|  | NT\_039472 |
|  | NW\_001030907 |
|  | Q2M4G5 |
|  | Q3TDN4 |
|  | Q3TIS9 |
|  | Q3TXE3 |
|  | Q3U447 |
|  | Q52KQ2 |
|  | 119604505 |
|  | 119604506 |
|  | 123984545 |
|  | 123998559 |
|  | 15559784 |
|  | 37504 |
|  | 42716354 |
|  | 56405328 |
|  | 62896903 |
|  | 74726316 |
|  | 123779493 |
|  | 123789998 |
|  | 123790567 |
|  | 123790886 |
|  | 123793140 |
|  | 148693212 |
|  | 148693213 |
|  | 18044031 |
|  | 23328874 |
|  | 33860233 |
|  | 5733095 |
|  | 62740256 |
|  | 62910180 |
|  | 74181753 |
|  | 74192938 |
|  | 74198577 |
|  | 74206622 |
|  | 81882293 |
|  | AI452085 |
|  | NW\_000351 |
|  | NT\_086897 |
|  | XP\_233741 |
|  | XM\_233741 |
|  | NW\_047798 |
|  | NC\_007305 |
|  | NW\_001495323 |
|  | XM\_590006 |
|  | XP\_590006 |
|  | XM\_002729874 |
|  | XP\_002729920 |
|  | NW\_001084867 |
|  | XM\_002726995 |
|  | XP\_002727041 |
|  | AABR03062309 |
|  | AABR03062407 |
|  | AABR03065697 |
|  | AABR03066411 |
|  | AAHX01053580 |

---

|  |  |
| --- | --- |
| Swiss-Prot ID | TYK2\_HUMAN |
|  | TYK2\_MOUSE |
|  | Q52KQ2\_MOUSE |
|  | Q3TXE3\_MOUSE |
|  | D3ZD03\_RAT |

---

|  |  |
| --- | --- |
| Cell Localization | Cytoplasm |

---

|  |  |
| --- | --- |
| Ensembl ID | ENSG00000105397 |
|  | ENSP00000431885.1 |
|  | ENST00000525621.5 |
|  | ENSP00000264818.6 |
|  | ENST00000264818.10 |
|  | ENSP00000433203.1 |
|  | ENST00000524462.5 |
|  | ENSMUSG00000032175 |
|  | ENSMUSP00000150354.1 |
|  | ENSMUST00000216874.1 |
|  | ENSMUSP00000150214.1 |
|  | ENSMUST00000214454.1 |
|  | ENSMUST00000213717.1 |
|  | ENSRNOG00000032948 |
|  | ENSRNOP00000048018.3 |
|  | ENSRNOT00000041678.5 |
|  | ENSMUSP00000001036 |
|  | ENSRNOT00000041678 |
|  | ENST00000525621 |
|  | ENST00000524462 |
|  | ENSP00000264818 |
|  | ENSRNOP00000048018 |
|  | ENST00000264818 |
|  | ENSMUST00000001036 |
|  | ENSP00000431885 |
|  | ENSP00000433203 |

---

|  |  |
| --- | --- |
| MGI ID | MGI:1929470 |
|  | 1929470 |

---

|  |  |
| --- | --- |
| RGD ID | 2320469 |
|  | 1307164 |

---

|  |  |
| --- | --- |
| Unigene ID | Mm.450004 |
|  | Hs.75516 |
|  | Rn.38688 |
|  | Mm.20249 |
|  | Rn.140313 |
|  | Bt.23803 |
|  | Rn.202161 |

---

|  |  |
| --- | --- |
| Homologene ID | 20712 |

---

|  |  |
| --- | --- |
| Shape | Sickle-vertex |

---

|  |  |
| --- | --- |
| IPI ID | IPI00022353 |
|  | IPI00653270 |
|  | IPI00359820 |

---

|  |  |
| --- | --- |
| Microarray ID | 97573\_at |

---

|  |  |
| --- | --- |
| Homo sapiens Chromosome position | 19p13.2 |

---

|  |  |
| --- | --- |
| Ensembl Transcript ID | ENST00000264818 |
|  | ENST00000525621 |

---

|  |  |
| --- | --- |
| FunctionalClass | ATP binding |
|  | Janus kinase |
|  | non-membrane spanning protein tyrosine kinase |
|  | nucleotide binding |
|  | transferase |
|  | kinase |
|  | protein kinase |
|  | protein-tyrosine kinase |
|  | protein serine/threonine kinase |

---

|  |  |
| --- | --- |
| Bos taurus Chromosome position | 7 |

---

|  |  |
| --- | --- |
| Mus musculus Chromosome position | 9 A3 |

---

|  |  |
| --- | --- |
| KEGG pathway | Adipocytokine signaling pathway |
|  | Jak-STAT signaling pathway |

---

|  |  |
| --- | --- |
| Rattus norvegicus Chromosome position | 8q13 |

---

|  |  |
| --- | --- |
| Ensembl Gene ID | ENSG00000105397 |

---
